# A mixed-methods study on perceptions towards use of Rapid Ethical Assessment to improve informed consent processes for health research in a low-income setting

**DOI:** 10.1186/1472-6939-15-35

**Published:** 2014-05-02

**Authors:** Adamu Addissie, Gail Davey, Melanie J Newport, Thomas Addissie, Hayley MacGregor, Yeweyenhareg Feleke, Bobbie Farsides

**Affiliations:** 1 Brighton and Sussex Medical School, Falmer, UK; 2 Addis Ababa University, Addis Ababa, Ethiopia; 3 Institute of Development Studies, University of Sussex, Brighton, UK

**Keywords:** Rapid ethical assessment, Ethiopia, Research ethics, Consent

## Abstract

**Background:**

*Rapid Ethical Assessment (REA)* is a form of rapid ethnographic assessment conducted at the beginning of research project to guide the consent process with the objective of reconciling universal ethical guidance with specific research contexts. The current study is conducted to assess the perceived relevance of introducing *REA* as a mainstream tool in Ethiopia.

**Methods:**

Mixed methods research using a sequential explanatory approach was conducted from July to September 2012, including 241 cross-sectional, self-administered and 19 qualitative, in-depth interviews among health researchers and regulators including ethics committee members in Ethiopian health research institutions and universities.

**Results:**

In their evaluation of the consent process, only 40.2% thought that the consent process and information given were adequately understood by study participants; 84.6% claimed they were not satisfied with the current consent process and 85.5% thought the best interests of study participants were not adequately considered. Commonly mentioned consent-related problems included lack of clarity (48.1%), inadequate information (34%), language barriers (28.2%), cultural differences (27.4%), undue expectations (26.6%) and power imbalances (20.7%). About 95.4% believed that consent should be contextualized to the study setting and 39.4% thought REA would be an appropriate approach to improve the perceived problems. Qualitative findings helped to further explore the gaps identified in the quantitative findings and to map-out concerns related to the current research consent process in Ethiopia. Suggestions included, conducting REA during the pre-test (pilot) phase of studies when applicable. The need for clear guidance for researchers on issues such as when and how to apply the REA tools was stressed.

**Conclusion:**

The study findings clearly indicated that there are perceived to be correctable gaps in the consent process of medical research in Ethiopia. REA is considered relevant by researchers and stakeholders to address these gaps. Exploring further the feasibility and applicability of REA is recommended.

## Background

With research increasingly being undertaken in low-income settings, there is a need to contextualize the application of ethical standards. Informed consent is one of the cornerstones of ethics in medical care and in health research irrespective of culture and geography. However, as elsewhere, the consent process in low-income settings is subject to influence from cultural beliefs and values. Experts in the field have emphasized that the challenges associated with research ethics in these settings are complex and cannot be addressed by regulatory processes alone. There is a need to move beyond guidelines and mere procedural ethical review to a broader system which is open to addressing the various other determinants in the developing world [1,2]. It is desirable to ensure that community members are involved in the local application of universal ethical values [3]. Assessment and monitoring of the process of informed consent are essential and are the joint responsibility of the local ethics review committee and the research sponsors. While ethics review committees can give oversight, only an active and transparent partnership between the research sponsor, investigators and the community can allow for effective on-going governance [1]. Whilst the concept and application of the doctrine of informed consent should be standardized and applied in the same way in any setting, the process of seeking consent quite appropriately varies. Amongst other considerations the culture of the people approached must be understood when balancing principles of individual autonomy against community-wide decision-making [4]. It has been argued that there is leeway for researchers to consider culturally-relevant strategies for obtaining informed consent from participants. Accordingly, researchers working collaboratively with local investigators and communities should be creative in designing approaches to acquiring informed consent in particular cultural environments [5].

Participatory action research methods such as Rapid Ethnographic Assessments are often employed to understand and address context-specific issues pertaining to cultural differences. They will almost always produce results in a fraction of the time and at lower cost than traditional qualitative research [6]. Farsides and Bull, in their study in The Gambia, developed an approach in which a form of rapid ethnographic assessment was carried out among key stakeholders, and the results were used to inform the design of the consent process for the studies in question. This approach has been termed *Rapid Ethical Assessment (REA)*, and has been piloted in two countries; a TB case-contact study and a vaccine trial in The Gambia, and a study on the genetics of podoconiosis in Ethiopia [7-10]. Other researchers have also applied the tool prior to genetic research in Ghana [11].

REA is a brief qualitative intervention designed to map the ethical terrain of the research setting prior to a research team recruiting participants. The model attempts to discover, describe and respond to the ethical issues specific to a particular research setting, and as such should help researchers to address the issues that genuinely matter to proposed participants. The enquiry is linked to the particulars of the research being conducted. For example, a study involving collection of blood samples would be preceded by an assessment of the ethical beliefs and attitudes pertaining to blood, bodily integrity, storage and use of bodily materials alongside a more general enquiry into the understanding of research, the standing of the research team, the local health economy etc. in the community being recruited. As such an REA serves the purpose of connecting ethical principles to contexts and realities on the ground. Its methodology employs a constellation of action research, rapid assessment and ethnography. The average duration of the REA process is 6 weeks, and is performed by an interdisciplinary team composed of subject area researcher, a social scientist and one or more local area experts. The assessment is conducted among key community stakeholders such as potential study participants, community leaders, and field and community workers to inform and guide the research consent process [8-11].

Since REA is a novel approach, it is important to generate convincing evidence on whether it is needed, acceptable and feasible on a larger scale. Even though research has documented the importance of such tools in low-income settings, there is no evidence regarding the feasibility of the approach on a larger scale, were it to be integrated into the existing research appraisal system. If the tool is to be recommended as a routine tool it must be feasible and practical in application. One element of this feasibility is the perceived relevance and acceptability of REA to researchers and research ethics reviewers. It has been assumed that the approach will be well received by the research community and gain acceptance, but this has not been tested. There is therefore a need to assess how REA is perceived by a range of research stakeholders including researchers, ethics committee members and policy makers.

In Ethiopia, a country with significant cultural, religious and linguistic diversity, recently there has been enormous expansion in education at graduate and post-graduate levels. Consequently, the number of health-related research projects has increased greatly over the past decade. These factors make it an ideal place in which to explore new approaches to the ethics of health research. An assessment was performed within the Ethiopian health research community, to explore their perceptions of gaps in the consent process and gather their opinions on the role of REA in improving the consent process.

## Methods

### Study area

The study was conducted between July and September 2012, in four major Ethiopian health research centres: Addis Ababa University (AAU), the Ethiopian Health and Nutrition Institute (EHNRI), Jima University (JU) and the University of Gondar (UoG). These institutions are prominent Ethiopian centres for health-related research with experienced staff covering a range of health-related disciplines.

### Study design and sample-size

A mixed methods approach employing both quantitative and qualitative data collection methods with a sequential explanatory design was used [12]. We included researchers, ethics committee members and research policy makers primarily working in the four institutions. The total sample size required for the quantitative study was 270 individuals. This was determined using a single population formula, with unknown proportion and 90% confidence limits. The sample size for the in-depth interviews was guided by the degree of information saturation based on preliminary analysis during data collection. Accordingly, a total of 19 interview participants were purposively selected.

### Data collection

Quantitative data were collected using a web-based (on-line), self-administered questionnaire backed by a paper based interview. The questions were structured and possible responses pre-coded. One open-ended question was included at the end of the questionnaire. The questions were designed based on variables identified through literature reviews including previous work on REA. The questionnaire was pre-tested by administering 15 questionnaires to researchers and academic staff at Saint Paul’s Millennium Medical College which is one of the new public medical schools in Ethiopia located in the capital, Addis Ababa. Based on the pre-test, the contents and sequence of some of the questions were revised for coherence and logical flow.

For the on-line data collection, e-mail addresses of researchers were obtained from department heads, institutional mailing lists, institutional web-sites and Institutional Review Boards (IRB) of the four institutions. Through the institution contacts we identified lists of all eligible respondents including researchers and academics with experience of independent research and a post-graduate qualification Exceptions were made to the second criteria when the researcher has many years of research experience. A total of 458 eligible participants were identified – 175 from AAU, 108 from JU, 99 from EHNRI, and 76 from UoG. The sample size (270) was proportionally distributed to the four centres. Respondents were randomly selected from the e-mail list using numbers generated by the RANDBETWEEN function in Excel (Microsoft Office 2007®). A link to the web-based questionnaire using SurveyMonkey® [13] was sent via e-mail to all randomly selected eligible respondents in AAU (103), JU (64), EHNRI (58) and UoG (45). A reminder was sent after 2–3 weeks to those who did not respond to the first e-mail invitation. The on-line survey stayed open for 45 days, and researchers who were not able to fill the on-line survey but preferred paper-based interviews were offered a printed questionnaire. The assistant data collector distributed the questionnaires and collected them at the end.

Qualitative in-depth interviews (IDIs) were used for collecting qualitative data. A question guide for the IDI was finalized after the preliminary analysis of the quantitative survey. The guide included questions around the existing gaps in research ethics, the ethics review process and the informed consent process. Feedback on REA based on further descriptions given by the interviewer, was sought. A total of 19 key informants were interviewed in order to gather more explanatory opinions and deepen information complementing the quantitative findings. Researchers, Research Ethics Committee members and administrators were interviewed. Interviews were conducted by the principal investigator (AA), either in English or Amharic, based on the convenience of the respondent. All interviews were digitally recorded. They were transcribed and translated to English by the research assistant (TA) and back translated by the principal investigator (AA).

### Data analysis

The SPSS-based quantitative data summary was downloaded from SurveyMonkey® [13]. Data cleaning and descriptive analysis was performed using SPSS for variables of interest. Responses to the in-depth interviews and the one open-ended question in the on-line survey were analyzed as text and thematically summarized. NVivo9® [14] was used to organize the data. Data were double coded to verify inter-coder reliability. Interpretations were drawn by triangulating both qualitative and quantitative findings.

### Ethical considerations

The research was approved by the Institutional Review Board of the College of Health Sciences, Addis Ababa University in Ethiopia and the Research Governance and Ethics Committee at Brighton and Sussex Medical School in the UK. After providing information about the research, consent was obtained from all study participants. The authors declare no conflicts of interest. The research was supported by the Wellcome Trust through Biomedical Ethics Doctoral Fellowship 089769.

## Results

After excluding incomplete responses, analysis was conducted on 241 complete responses, which was 89.3% of the intended sample. The majority of the respondents were male (86.3%) and in the 26–35 year old age group (42.7%). Most (82.2%) were from the four target institutions AAU, EHNRI, JU and UoG; the rest were from the Armauer Hansen Research Institute (AHRI) the Federal Ministry of Health (FMOH), the Ministry of Science and Technology (MoST) or the Ethiopian Public Health Association (EPHA), who also were collaborators on research projects with the four main target institutions. More than sixty percent of respondents were either medical or public health professionals and 87.1% had postgraduate or specialty medical education, either Master’s degree, clinical residency or PhD; the rest had only undergraduate training with or without post graduate diploma [Table 1].

**Table 1:** Socio-demographic profile of respondents to the online survey, September, 2012 (n = 241)

**Variable**	**Frequency**	**%**
**Sex**		
Male	208	86.3
Female	33	13.7
**Age**		
<26	19	7.9
26-35	103	42.7
36-45	71	29.5
46-55	39	16.2
>55	9	3.7
**Institution**		
Addis Ababa University	87	36.1
Jimma University	49	20.3
EHNRI	35	14.5
University of Gondar	27	11.2
Others (MOH, MOST etc.)	43	17.8
**Highest qualification**		
Bachelor	20	8.3
MD/DVM	14	5.8
Masters	129	53.5
Specialty	32	13.3
PhD	35	14.5
Others (e.g. PGD)	11	4.6
**Major training** (multiple responses)		
Biology	28	11.6
Public Health	90	37.3
Social Science	13	5.4
Medicine	63	26.1
Laboratory	33	13.7
Nursing	19	7.9
Others (e.g. Environmental health, health promotion etc.)	63	26.1
**Roles in research** (multiple responses)		
Lead researcher/ PI	198	82.2
Co-investigators	179	74.3
Data collector	92	38.2
Field Worker	76	31.5
Ethics committee member	62	25.7
Data encoder	23	9.5
Other	19	7.9
**Training on research ethics**		
Yes	158	65.6
No	83	34.4

Most respondents assumed roles as principal investigators (82.2%), or co-investigators (74.3%). About 70% had also taken roles as data collectors or field workers (69.7%) and one quarter had been members of an Ethics Committee. More than a third (34.4%) reported that they had never had any training on research ethics [Table 1]. The types of training courses attended by trained respondents included orientation sessions (36.1%), certified short training courses (43.7%), academic training courses (university courses including diploma and degree level training) (37.3%) and other training courses (such as on-line) (11.4%).

Nineteen researchers, faculty, IRB members and policy makers from AAU, EHNRI, JU and MoST responded to the qualitative study. The majority (14) were male. All the respondents had experience working in various places in Ethiopia. All except one had post-graduate qualifications; the exception was an experienced IRB administrator at AAU.

### Perceptions about current consent processes and the relevance of REA

A majority of survey respondents (58.9%) believed that the design and preparation of consent processes for research was conducted by the principal investigator without any prior assessment of potential context-specific ethical issues and only 15.4% claimed to be satisfied with the current process by which consent forms are developed and implemented. Some respondents mentioned forms of prior assessment, including stakeholder or sponsor consultations, as shown in Table 2. When asked about their personal evaluation of the current consent process, only 14.5% thought that the best interests of the study participants were sufficiently considered; and only 40.2% thought the consent information and the consent process were adequately understood by the study participants. The most frequently reported challenges in the consent process were; lack of clarity of the information contained in the information sheet (48.1%), inadequate information (34%), inappropriate language and terminologies used (28.2%), cultural differences (27.4%), undue expectations (26.6%), power imbalances (20.7%) and coercion (7.9%) [Table 2]. The IDI respondents also mentioned gaps such as; language issues, lack of awareness, undue expectations and manipulations by researchers.

**Table 2:** Opinions of researchers from various Ethiopian institutions on the consent processes and development of REA, September, 2012 (n = 241)

**Variables**	**Frequency**	**%**
**How is consent form developed in your experience?** (multiple responses)		
By the investigator	142	58.9
With prior ethical assessment	70	29.0
With stakeholder participation/consultation	41	17.0
By the sponsor	17	7.1
Others (research advisors, collaborators, students)	13	5.2
**Opinions on consent process**		
Do you think all participants understand consent forms well?		
Yes	97	40.2
No	144	59.8
Based on your experiences, are you satisfied with the way the consent process was designed and conducted? (Y/N)		
Yes	37	15.4
No	204	84.6
Do you think that the best interest of study participants is taken in to consideration and adequately addressed through the current ethical appraisal and consent processes?		
Yes	35	14.5
No	206	85.5
Based on your experiences, what do you think are the most common problems in the current consent process?		
Lack of clarity	116	48.1
Inadequate information	82	34
Language	68	28.2
Cultural difference	66	27.4
Undue expectations	64	26.6
Power imbalance	50	20.7
Coercion	19	7.9
Others	11	4.6
**Ethical Pre–assessment for Consent Process**		
Do you think it is important to contextualize consent forms and consent processes to local settings?		
Yes	230	95.4
No	11	4.6
Do you agree with the idea of the study participant be approached in advance before the start of the study to get input for the development of the consent form and to find out how it should be administered?		
Yes	196	81.3
No	45	18.7
Do you think that study participants should be involved, in the development of consent forms and designing of the consent process so as to make it culture and setting sensitive?		
Yes	171	71
No	70	29
In your opinion, would REA serve adequately addressing the consent process issues and in making sure that ethical issues are very well addressed in a research process?		
Yes	95	39.4
No	146	60.6
From your experiences, is there any initiative so far; is there any initiative that involves the study participants in the development and design of consent information sheet and consent process?		
Yes	29	12
No	212	88

#### Language

The problem of language was repeatedly mentioned as a challenge to the consent process. In Ethiopia it is assumed by researchers that Amharic, the national language, will be used all over the country and can be used for the purposes of obtaining consent. Respondents indicated that the country is rather diverse and multi-ethnic, where there are many other languages and dialects.^a^ Failure to understand differences may be problematic. According to the national guidelines, all consent documents in a research protocol need to be translated into the national language, irrespective of the language of data collection which may not be Amharic. This is one of the points highlighted in the checklists of ethics committees and IRBs. A mechanism ensuring local language and appropriate versions of translations are used in the field is lacking. In certain societies, the terms and concepts of research and most medical terminologies do not exist. In addition, concepts of health and disease may be based on local traditional understandings rather than the modern medical models used by researchers.

*“When we come to information, there is a problem of language. … Ethiopia is a diverse country, so even when you translate to Amharic*^b^*, it is hard to bring the understanding. It is hard to translate scientific words to Amharic. …”* [Researcher, AAU].

#### Lack of awareness about research, health and ethics

Generally there is little awareness or understanding of science and research, especially in those with very low levels of literacy. Thus, participants might consent or decline based on their lack of comprehensive understanding about the research process.

“The society might be where they have no knowledge about research. … People might also not understand what is written if they can’t read. These are [some] loop holes” [Researcher, EHNRI].

*“Even, the participants don’t know what … research is … They don’t know whether the research has benefit or harm … [they] say yes without knowing it*” [ Researcher, JU].

“.......*In Ethiopia, [in] most places where research is done, the people don’t know how to read and write, so how much do the researchers need to explain?”* [*Researcher,* AAU].

#### Undue expectations by participants and manipulation by researchers

Due to misconceptions and failure to comprehend the intended purpose of research, especially in rural communities, there is a tendency to expect direct benefits. Undue promises may be given by field workers just for the sake of obtaining consent.

*“… (even with) the tone of your voice, you might emphasis mainly the benefit, there might be some persuasion. [And]), they (participants) have to believe in it”* [Researcher, JU].

#### Focus on consent and recruitment

There is a tendency to focus more on the consent i.e. the decision to participate, than on ‘informed consent’ which should be based on prior information and comprehension. As long as consent is given it is considered alright to negotiate and bargain.

*“ … there is a lot of negotiation and bargaining (around consent), which is not a proper way of consent (process) application”* [Researcher, JU].

#### Emphasis on rules and procedures

Respondents felt that there was too much emphasis on fulfilling requirements and procedures rather than on genuine concern for the rights and welfare of study subjects. Researchers cannot proceed without fulfilling these requirements and for most researchers ‘research ethics’ is about completing paperwork to get the ‘ethical clearance letter’. The regulators also focus more on the enforcement of the rules without also creating awareness among researchers and the research community. Most regulators do not explain the reasons why the review is needed. The undue emphasis on rules and procedures has created discouragement on the researchers’ side. Researchers expressed their concern that too much emphasis on the rules and procedures my eventually discourage and hamper the progress of research and science.

*“Even the Civil law (code) has been too protective, to the extent of not allowing the conduct of trials – Ethics should not hamper Science!”* [Researcher, AAU].

### Suggestions to improve the current consent process

Based on the open-ended questions, the following major thematic areas were suggested by the survey respondents as ways of improving consent process.

#### Involvement of the community and potential research participants

Respondents suggested that the community needed to be involved in the research process in various ways such as by including community representatives in ethics committees, and by seeking the opinions of community leaders on potential ethical issues prior to and during research. Suggested mechanisms included approaching and involving the community through Community Advisory Boards (CAB).

#### Training

Various types of ethics training courses were also suggested by the respondents as important avenues for improving the consent process in developing countries. The training courses might be for researchers, health professionals or the general public. Suggestions were given on how the training courses might be delivered. These included integrating the course into research methodology courses and making them part of undergraduate and postgraduate degrees.

#### Differentiated approach – depending on the type of research

Participants emphasized the importance of acknowledging differences in contexts and designing approaches accordingly. Using REA depends on prior knowledge of the research design and anticipation of emerging ethical issues. Approaches would depend on the type of research design and the sensitivity of the topic to be addressed by the research, for example clinical trials in which biologic specimen collection takes place.

#### Pre-assessment

Many appreciated the idea of REA as a way of performing an assessment prior to the actual field study. Involving potential study participants in the assessments was also suggested, while others suggested it would be better to involve the CAB rather than study participants. (See theme ‘involvement of community’ above).

#### Field visits

Field visits were suggested, to follow the way in which ethical procedures stated in the protocol were actually being implemented in the field. These visits might be before the study or after the study, to monitor the implementation of consent or any emerging issues, depending on the nature of the study. The mandate of deciding which projects needed the field follow-up, and coordinating follow-up, would be that of the IRB.

#### Balanced approach, ‘not to be a hurdle for research’

Out of concern that too much emphasis on rules and regulations might discourage researchers, respondents mentioned how important it was that ethical regulations did not become a barrier to the research itself. The importance of striking the correct balance was also reflected in informal discussions with researchers.

### Perceived relevance and feasibility of REA

Regarding the importance of conducting a pre-assessment to explore potential context specific ethical issues for designing the consent form, 95.4% of the survey respondents agreed it was important to contextualize consent to the setting; 81.3% thought it was important to approach participants before the study to get input into the consent process; 71% thought it was important to involve local people in consent design in some way; 39.4% agreed to the idea of doing an additional and separate rapid assessment of the local situation such as REA before designing the consent process, with just 12% reporting that they already knew of such initiatives. On further inquiry, the following reflections on the relevance of REA were made by researchers and research ethics committee members during IDIs.

#### Need for strengthening the existing research ethics review system

Participants mentioned that ethics review in Ethiopia is a relatively new phenomenon. The system is not very uniform and relies on the experience of very few individuals. The national guidelines were thought to be old and ripe for review. Thus the need for rigorous and tailored ethics review was mentioned. Respondents emphasized the need for addressing research ethics in proportion to the expansion in health research in the country.

#### Opinions on the current consent process and consent review process

The consent process is mainly dealt with by individual researchers who in most cases have very little experience or training in research ethics. At times, they may seek counsel from experts. As a result, there is a tendency to perceive ethics review as another bureaucratic step rather than for the scientific and moral merits it may have. Several researchers perceived ethical review as discouraging, as a mere administrative matter. On the other hand, respondents agreed that there needed to be review of certain types of research but not for all research. Most were sceptical about the amount of time it takes for review of simple projects such as secondary data analysis. Coming to the review process, this is done based on what is stated in the protocol, but what happens in the field is not known – there is usually no check on comprehension. Authors of this paper have had similar experiences both as members of IRBs for over two years and as researchers getting ethics approval for research projects. In the IRB meetings, the focus is more on the way the proposal is written and the informed consent designed and written. The only check point closer to the community is the presence of a lay person. The lay person reads all the information sheets during the IRB meetings. Language being one important issue, the lay person usually checks the English version of the information sheet and consent form.

#### Rapid ethical assessment

Conducting a prior assessment at the beginning of a study with the aim of exploring context-specific ethical issues was considered a good idea by most, but there were concerns surrounding practicality and feasibility. Based on the descriptions given to them of REA, the majority appreciated the tools. However, there was confusion around the term ‘rapid’. Many thought that this was about a ‘rapid’ ethics *approval* process. Further explanation had to be given about the term ‘rapid’ in REA, that it is a method of assessment used to explore the ethical issues in a relatively quick fashion at the beginning of the study and has nothing to do with speeding up the process of ethics review. Once they had understood the concept and the implied purposes, most respondents saw REA as a tool that would enhance the community’s role as a stakeholder in research and would address the community’s concerns with regard to ethical issues arising during research. There was agreement among most that in any research, the community needed to be contacted to seek counsel ahead of time regarding their concerns including ethical aspects of the research projects.

*“… you don’t go to the field with your backpack and say I am from France, and assuming these people are subjects and they will agree, this is what was done in the old days. Now in the community, you have to try to teach them as much as possible, telling them the pros and cons of the research. … They have to negotiate their benefit”* [ *Researcher, AAU*].

*“Things that are important to the local people might not be important to the others, so to make it relevant to the local people, we need to ask questions like ‘do you have any concerns?’, ‘do you have a question?’ at the piloting time. …collecting this questions and [then] incorporating (addressing) them (the concerns)”.* [*Researcher, JU*].

#### Issues to be taken into consideration for REA

Asked whether they would recommend the REA tool for all research projects, many indicated that the approach would depend on the type of research. As REA takes time and additional resources, it might not be feasible to apply it to all research projects. Most mentioned this need to be dictated by the nature of the study. The existence of prior knowledge about the community was suggested as an important issue. If the ethical issues in the setting are already mapped out, there may not be a need to do REA.

*“Yes for big research studies, for example clinical trials … (and for) cohort studies …it might be possible. … Around here most research projects are cross-sectional, … the days of data collection might only take 10 days. If you ask them to do the consent form [based on REA] and if it takes them 3 months, it might not be practical for most people. But for bigger projects, I think it will have a place…”* [*Researcher, JU*].

“*If we already know [the ethical issues in] our own community there is no use in doing an additional formative assessment.” *[Researcher, JU].

## Discussion

Irrespective of the final conclusions relating to REA, the study highlighted important considerations for improvements in the research ethics system in Ethiopia. Whilst there have been a number of efforts at institutional and national levels to build a competent ethics governance and review system, the views of stakeholders suggest that further work is required. Given the relatively narrow range of individuals surveyed (institutional researchers and regulators), it could be claimed that more comprehensive assessment of the research ethics governance system in Ethiopia is needed before making further remarks. The study also identified clues specifically related to the research consent process in Ethiopia both at information provision and decision-making stages. The findings suggested that REA tools could be considered relevant and potentially feasible in the Ethiopian context in order to address these gaps.

The on-line survey for the quantitative component, which we believe to be one of the first uses of this technique in Ethiopia for health research, was efficient in terms of generating information rapidly. However, researchers who were not accessing their e-mails during the data collection period were not included in the study. On the other hand, a significant proportion of the intended sample size was reached, as respondents were able to answer the survey questions irrespective of their physical availability or current location given internet connectivity. The fact that the respondents themselves were researchers who understood the importance of responding to surveys may have contributed to the high level of compliance [15,16]. It was possible to send reminders and additional invitations on the basis of responses. This may have resulted in selection bias as busy people and those on vacation during the survey might not have responded to the survey. However, we do not know reasons for non-participation and non-response. The study was conducted at the beginning of the academic year, assuming most academics would have time to complete an online survey. The study has demonstrated the potential of using on-line survey tools in Ethiopia among groups such as academics and researchers. The other advantage of conducting the on-line survey was the use of the preliminary findings to shape the qualitative study.

The mix of study respondents is representative of the group targeted for the study; high level researchers and regulators of ethics review systems with considerable research experience. Their current roles and experience in research made them ideal to identify gaps in health research ethics, and to suggest possible ways of addressing them. However, since the study was limited to researchers and regulators, the perspectives of other important stakeholders in health research such as non-researcher health professionals, community members, senior officials and country level policy makers were not included in the study. Another point to note about the respondents is that, despite the fact that most were involved in conducting research, in ethical review or in the development of ethics review guidelines, a significant proportion of respondents had not had any formal training in research ethics. Possible explanations for this include the absence of structured research ethics training courses and the fact that Ethiopia is in the early stages of implementing a universal system of research ethics. One example of this is the recommendation given by the study participants about having a community representative on ethics review committees. This is already included in the national ethics guideline [17], but is not uniformly implemented. This lack of standardized and structured ethics training is a critical gap, and from the qualitative findings, there are suggestions that it may lead to systemic gaps in the ethical conduct of research. Other studies have documented knowledge gaps among academics and have suggested ways of including ethics training in mainstream curricula [18,19]. In addition, the qualitative study did not include representatives of all academic and research institutions, which makes it difficult to generalize the findings to other settings. Yet we believe that most of the concerns are shared by similar institutions. The use of a mixed-methods design served to triangulate findings as the qualitative assessment provided deeper insight into the survey findings and bridged the gaps in the survey assessments. The online survey enquired, with closed options, into ethical pre-assessments and REA in reference to involvement of potential study participants, without further explanation of the REA concept. With the IDIs it was possible to discuss further, explain the essence of REA and enquire into the involvement of community members beyond study participants. Overall, the study was able to identify key issues relating to research appraisal and explore perceptions surrounding REA.

Respondents’ perceptions of existing consent processes were not found to be favourable. Most respondents thought that potential participants understood little about the consent process or the information provided to them. They also thought that participants’ best interests were rarely considered, reflecting gaps in communication and the decision-making process. The major gaps for study participants in relation to communication and comprehension included use of incomprehensible specialist terms to explain medical concepts, lack of health and research awareness of participants, undue expectations and manipulation by researchers. The same list of issues could influence decision making, which in turn is influenced by factors such as manipulation by researchers and the local dynamics in decision making. These factors vary from context to context. In Ethiopia ethno-cultural differences are very visible, and they make the use of generic consent forms and consent approaches for varied setting inappropriate.

Traditionally, consent forms have been developed by researchers based on the requirements of an information sheet and a decision page. This approach addresses consent from the perspective of the basic principles in ethics and the major international guidelines. The assumption is that the contents would be relevant fairly standardized irrespective of who the participants are. Sometimes consent forms have been developed by international investigators and ‘adapted’ purely by translation.

In Ethiopia, communities have varied levels of awareness of public health issues. According to the Ethiopian Demographic Health Surveys (EDHS), levels of understanding vary by characteristics such as area of residence, income, region, religion, ethnicity, and literacy status [20]. Tekola *et al.* reported that words for ‘health’, ‘research’ and ‘medical treatment’ either do not exist in some languages or are used interchangeably in a confused and confusing way [8]. This may give rise to expectations of medical treatment which confound the decision making processes. Some researchers might be tempted to take advantage of this vulnerability of participants to increase rates of recruitment by either promising unrealistic and unavailable benefits or at least by not challenging misconceptions. Gaps in the consent process are reported elsewhere related to information and communication and decision making and indicate the need for informed consent processes tailored to the context [1,8-10,21-26]. The use of qualitative assessment in the informed consent process for medical research in developing countries have also been reported [27].

Based on previous studies which have employed REA, consent processes have significantly improved in terms of both comprehension and decision making as a result of the knowledge and understanding gained and the steps taken to incorporate it. The key steps were identification of issues prior to and during the conduct of studies and guiding the consent process (including the provision of information) based on those qualitative findings. The REA tool has been recommended for use by researchers who have used them in their respective research [7-11]. The current qualitative findings support the need for pre-assessment to explore potential context-specific ethical issues. Pre-assessment in health research may have various objectives such as testing data collection tools and assessing study feasibility [28-32] and optimizing community engagement [33]. However REA is distinct from these forms of pre-assessment being focused primarily on ethical issues.

It is interesting that a significant proportion of the respondents mentioned they were aware of the role of some form of pre-assessment and stakeholder participation in exploring ethical issues. However, the extent of actual use of pre-assessment was not measured, and neither was any quantitative assessment made of the techniques that might already be used by researchers. The qualitative responses revealed that *ethical* pre-assessment are not widely used, and that the term may be used to refer to feasibility studies or piloting of tools. Genuine ethical pre-assessment is rare, and stakeholder participation usually refers to involvement of community representatives in identifying the problem to be investigated rather than the consent process *per se*, [34-36]. There are fewer experiences of pilot studies for ethical pre-assessment, and these were limited to the specific research projects under consideration and did not investigate the openness of researchers to their wider scale use [37-39].

Currently in Ethiopia, there is no established mechanism in place to assess the community risks, vulnerabilities and benefits beyond what is written in the application submitted for approval. Most IRB reviews are based on a simple Risk-Benefit assessment of the submitted proposal and an assessment of whether the consent form meets an acceptable standard and is written well. However, one important parameter in IRB formats is ‘Involvement of the community in the research’. Again, in practice this assessment of community involvement is based solely on what has been included in the proposal, and refers mainly to involvement in the development of the research question, which is understandable given that one of the implicit principles in community health research, is that ‘community concern’ is an important criterion.

A ‘pre-assessment’ which includes the community would reduce the reliance of the IRB on making a judgment based on the written scientific proposal alone. Community engagement approaches, which aim to create awareness and a sense of ownership by the community, are becoming more popular. These approaches often work with and through community groups such as Community Advisory Boards. Community engagement is also used as a process of influencing change in the community through provision of information, negotiation, local capacity building and empowerment [33,40]. Community engagement might also be used to address ethical issues around research [26,41-43]. Community engagement and REA share some overlaps, since they both address community issues and share qualitative methodological approaches. However, REA is primarily conducted by a REA team (a multi disciplinary team of researchers) and employs rapid ethnographic research methods, while community engagement will more commonly seek to involve community members and to engage over a longer period than suggested for REA. The two are not mutually exclusive.

The study documented considerable interest in REA as a tool to improve the research consent process. However, respondents were concerned about the potential burden REA would put on the researcher in terms of time and other resources. There is a concern that REA may unnecessarily delay small, cross-sectional research projects into less sensitive topics. One suggestion made was to conduct REA as part of the traditional pre-test during community based studies. However, the objectives and duration of such pre-tests vary considerably. Some are rapid and done in a day or two and would not permit REA, which required about 4–6 weeks in the earlier studies [8,10]. Other types of pilot studies such as feasibility studies for randomized trials would be ideal in terms of integrating REA. The studies so far employing the REA method have documented its significant contribution in identifying important ethical issues in the research context [10,11]. However, such studies have not assessed the usability of the tool by the research community. As REA is an additional tool which requires time and expertise, research teams need to take this into account when planning their project. It is then important to be clear about what resources are required so that researchers can take this into consideration. In addition, REA findings might possibly raise issues relevant to study design and therefore might best be done in advance of most pre-tests.

According to the findings of the current study, the ‘rapid’ aspect of the REA tool was at times confused with the idea of expediting the review process. Some respondents were confused about the terminology as they tended to understand that the tool was ‘rapid’ and was meant for accelerating the ethical review process. Most of the researchers indicated that the current appraisal process takes too long and there is a need for finding a way to improve this. They were intrigued to learn that REA entails additional time and resources.

Whether REA should be applicable to all studies depends on a number of issues such as the nature of the study, the characteristics of study participants, the nature of the issue under investigation and the availability of resources. Whilst it is important to avoid crude generalisations and stereotyping when setting ethical standards, it is possible to learn from similar cases. Sometimes earlier studies of a similar nature will provide information on which consent processes can be designed. Clinical trials and studies that include vulnerable subjects or sensitive issues would often require REA. In clinical trials and classical longitudinal studies, there are a number of encounters with the study subjects. This would allow plenty of room for addressing consent issues. Such longitudinal studies require repeated and long term encounters between researchers and the community. As the consent process is repeated, it can be improved. Some communities are better informed about science and research than others and are well known to the researcher. In localities where health systems are well accessed and previous research has been conducted, the community generally has a better understanding of health research and the anticipated ethical issues will already be familiar to researchers. REA is therefore likely best reserved for communities that are not well researched and are less familiar to researchers.

The main objective of the current study was to assess the attitudes of the research community towards the REA tool and their views on its perceived relevance. In this paper we did not intend to explore the issue of practical feasibility beyond recording the perceived relevance and applicability of the tools as expressed by researchers and research ethics reviewers. Perceived relevance of the tool is different from its practicality and feasibility which need to be explored further. Expressed demand and acceptability of an approach are important components in assessing feasibility but are not sufficient [44]. However this study has clearly demonstrated the openness of researchers to considering the introduction of such a tool and suggests that they might willingly collaborate in developing a feasible intervention.

### Limitations

Qualitative data analysis was done in English while conducting the analysis in the language of data collection would have been preferred. Community representatives were not included in the study and the assessment regarding the research ethics review system in Ethiopia was primarily based on perception of researchers and ethics committee members. We used post graduate training as a proxy indicator of research experience which may not always be correct as there are variations in the profiles of post graduate programs.

## Conclusion

REA tools and techniques were found to be highly relevant and acceptable to the Ethiopian research community, however practical challenges were anticipated in the actual implementation. Research ethics and its review systems are relatively new in Ethiopia, though the country has national guidelines and structures in place to regulate ethics in health-related research. Even though there is rapid expansion of health and medical research activities due to acceleration of post graduate education, there is a lack of capacity in ethics review systems, particularly in the newly-founded Universities. The current review process has demonstrated critical gaps in ensuring reliable consent processes. The problems arise from three distinct areas; those embedded in the health research review system, those related to researchers and those related to the general public. REA is not a panacea but the introduction of a manageable level of pre-assessment of research settings would provide researchers, ethics reviewers and policy makers, with a manageable amount of data relevant to all the issues raised here as challenging and/problematic in an Ethiopian context. For this reason the applicability and practical feasibility of REA needs to be further explored, and the openness of researchers to embracing this tool needs to be capitalized upon in the interests of future research participants.

## Endnotes

^a^Ethiopia is a country of over 80 million inhabitants with over 80 ethnic groups and more than 80 different languages spoken while Amharic is considered as the official working language.

^b^The Ethiopian official working language.

## Abbreviations

AAU: (Addis Ababa, University); AHRI: (Armauer Hansen’s Research Institute); CAB: (Community Advisory Board); EDHS: (Ethiopian Demographic Health Survey); EHNRI: (Ethiopian Health and Nutrition Research Institute); EPHA: (Ethiopian Public Health Association); FMOH: (Federal Ministry of Health); IDI: (In-depth Interview); IRB: (Institutional Review Board); JU: (Jimma University); KII: (Key-informant Interview); MoST: (Ministry of Science and Technology); REA: (Rapid Ethical Appraisal); UoG: (University of Gondar).

## Competing interests

The author(s) declare that they have no competing interests (financial and non-financial). The study was financially supported by Wellcome Trust (WT) through Biomedical Ethics Doctoral Fellowship 089769. However WT has no any part in the execution of the study and write-up.

## Authors’ contribution

AA, GD, and BF designed the study. MN, HM and YF critically evaluated and made progressive suggestions on the initial study design. AA developed study instruments and collected and organized data with TA. AA and TA did data collection and analysis including transcriptions, initial coding and double coding. All authors (AA, GD, BF, MN, TA, YF and HM) were involved in the write up of the manuscript and in the critical review of drafts. All authors read and approved the manuscript.

## Pre-publication history

The pre-publication history for this paper can be accessed here:

http://www.biomedcentral.com/1472-6939/15/35/prepub
